# Supplementary material for: Causal Relationship between Aging and Anorexia Nervosa: A White-Matter-Microstructure-Mediated Mendelian Randomization Analysis
Source: Biomedicines. 2024 Aug 16;12(8):1874. doi: 10.3390/biomedicines12081874 (PMC11351342; doi:10.3390/biomedicines12081874)
Supplement: Supplementary file 1 [file biomedicines-12-01874-s001.zip › Supplementary Figure/Figure S4/Singe SNP plot of MR analysis for TL to ubm-b-1888.pdf]

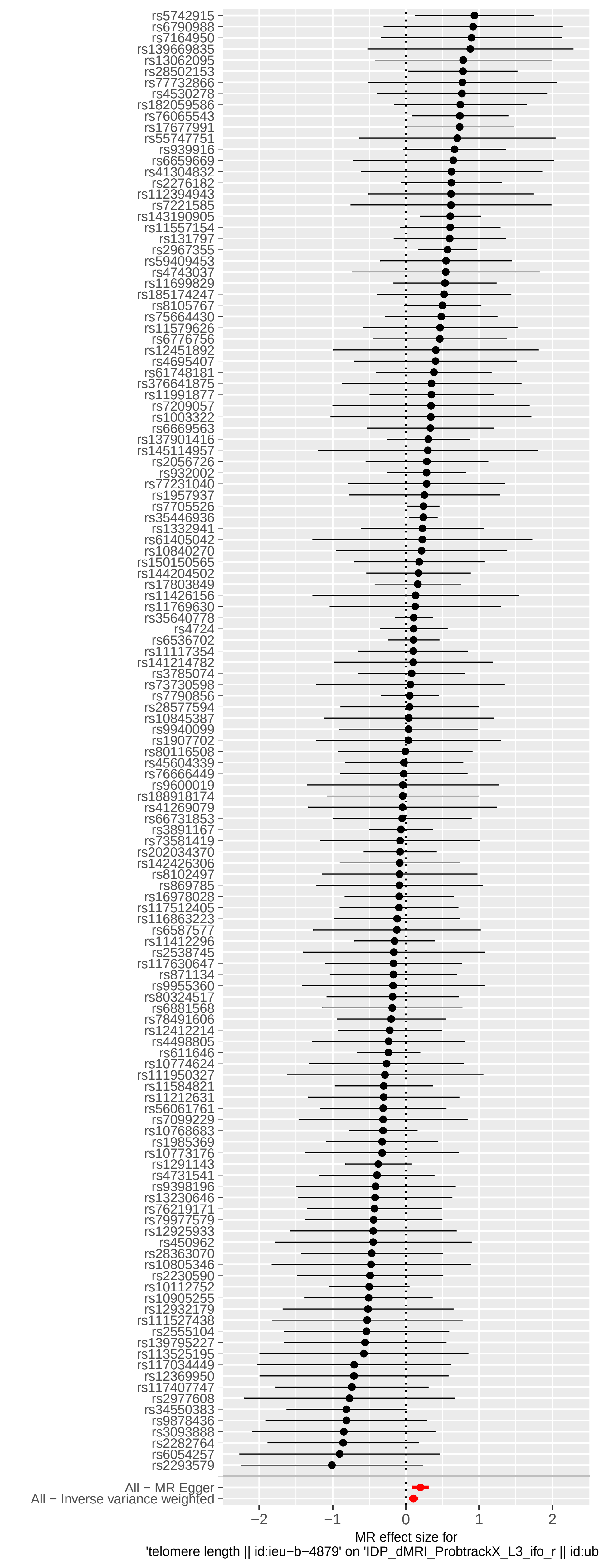

rs5742915
rs6790988
rs7164950
rs139669835
rs13062095
rs28502153
rs77732866
rs4530278
rs182059586
rs76065543
rs17677991
rs55747751
rs939916
rs6659669
rs41304832
rs2276182
rs112394943
rs7221585
rs143190905
rs11557154
rs131797
rs2967355
rs59409453
rs4743037
rs11699829
rs185174247
rs8105767
rs75664430
rs11579626
rs6776756
rs12451892
rs4695407
rs61748181
rs376641875
rs11991877
rs7209057
rs1003322
rs6669563
rs137901416
rs145114957
rs2056726
rs932002
rs77231040
rs1957937
rs7705526
rs35446936
rs1332941
rs61405042
rs10840270
rs150150565
rs144204502
rs17803849
rs11426156
rs11769630
rs35640778
rs4724
rs6536702
rs11117354
rs141214782
rs3785074
rs73730598
rs7790856
rs28577594
rs10845387
rs9940099
rs1907702
rs80116508
rs45604339
rs76666449
rs9600019
rs188918174
rs41269079
rs66731853
rs3891167
rs73581419
rs202034370
rs142426306
rs8102497
rs869785
rs16978028
rs117512405
rs116863223
rs6587577
rs11412296
rs2538745
rs117630647
rs871134
rs9955360
rs80324517
rs6881568
rs78491606
rs12412214
rs4498805
rs611646
rs10774624
rs111950327
rs11584821
rs11212631
rs56061761
rs7099229
rs10768683
rs1985369
rs10773176
rs1291143
rs4731541
rs9398196
rs13230646
rs76219171
rs79977579
rs12925933
rs450962
rs28363070
rs10805346
rs2230590
rs10112752
rs10905255
rs12932179
rs111527438
rs2555104
rs139795227
rs113525195
rs117034449
rs12369950
rs117407747
rs2977608
rs34550383
rs9878436
rs3093888
rs2282764
rs6054257
rs2293579
All − MR Egger
All − Inverse variance weighted
−2
−1
0
1
2
MR effect size for
'telomere length || id:ieu−b−4879' on 'IDP_dMRI_ProbtrackX_L3_ifo_r || id:ub
